# The differences between normal and obese patient handling: re- structural analysis of two questionnaires

**DOI:** 10.1186/s12891-023-06479-7

**Published:** 2023-05-06

**Authors:** Rashid Heidarimoghadam, Saeedeh Mosaferchi, Pradip Kumar Ray, Hamid Saednia, Khadijeh Najafi Ghobadi, Alireza Mortezapour

**Affiliations:** 1 Health Sciences Research Center, Department of Ergonomics, School of Public Health, Hamadan University of Medical Sciences, Hamadan, Iran; 2 Department of Industrial Engineering, University of Salerno, Fisciano, Salerno, Italy; 3 Department of Industrial and Systems Engineering, Indian Institute of Technology Kharagpur, Kharagpur, 721302 India; 4 Department of Ergonomics, School of Public Health, Hamadan University of Medical Sciences, Hamadan, Iran; 5 Department of Biostatistics, School of Public Health, Hamadan University of Medical Sciences, Hamadan, Iran

**Keywords:** Patient transfer, Musculoskeletal diseases, Moving and lifting patients, Patient safety, Validation study

## Abstract

**Background:**

Precise causes of musculoskeletal complaints among nurses are not known well, but many studies have pointed to manual patient handling tasks. Subjective judgment and decision-making process for patient lifting is crucial for gathering data regards patient handling. The aim of this study was to consider reliability and validity and re-structure of two special tools for patient handling’s tasks.

**Methods:**

In this cross- sectional study 249 nurses were fully participated. As recommended by literature for cultural adaptation of instruments, forward/backward translation method was applied. Reliability of the translated version was assessed by Cronbach’s alpha coefficient. Validity testing for the two scales was based on content validity index/ratio analysis and also Exploratory Factor Analysis was run to extract latent factors.

**Results:**

Reliability estimated by internal consistency reached a Cronbach’s Alpha of above 0.7 for all subscales of two questionnaires. After testing the validity, the final version of questionnaires was remained by 14 and 15 questions respectively.

**Conclusions:**

These instruments evaluated for manual handling of normal and obese patients had acceptable validity and reliability in Iranian Nursing context. So, these tools can be used in further studies with the same cultures.

## Background

Musculoskeletal disorders (MSDs) are known as a crucial problem that nurses must endure it because of their duties [1, 2]. This fact also was pointed out in recent original [3, 4] and literature review [5] studies. The precise causes of musculoskeletal complaints among nurses are not known well [6] but many studies have pointed to manual patient handling tasks [1, 7]. Maybe that is why the safe transferring and handling patients have remained as an ergonomic concern in healthcare systems worldwide [8, 9].

It is true that complete avoidance of manual patient handling was a crucial health recommendation for nurses [1], but manually ambulating and repositioning patients occurs frequently in daily work activities of nurses and caregivers [2, 3]. Nowadays it is well-believed that manual patient-handling is a physically demanding task [1, 2] and that is accounted for up to 72% of MSDs cases among hospital workers [7, 10]. When nurses manually handle patients, the recommended maximum compression force which was advised by The National Institute for Occupational Safety and Health (NIOSH) of the United State of America can easily reach (3400 Newtons). But when appropriate patient-handling equipment is used the physical burden might be reduced to a safe level [11, 12]. Elnitsky has showed that with positioning, lifting and transferring patients, nurses experience high prevalence of MSDs [13]. Also results of a large prospective cohort study demonstrated that nurses with daily patient-handling tasks had the more risk of MSDs problems in comparison to nurses without daily patient-handling [14].

Indeed, it is true that accessing to a variety of patient-handling instruments, good ergonomic intervention and training may decrease the chance of nurses’ physical health problems [15–17], but studying manual patient handling is still a challenging topic [18]. So it can be claimed that in addition to objective studies, the study of manual handling of patients according to the subjective data can also have an effective role in improving nurses’ working conditions [19, 20]. In patient handling duties based on the patient body mass, awkwardness and unpredictable nature of the task and environment, subjective judgment of nurses and health professionals about patient handling task must be considered [21, 22]. For this reason in some studies nurses’ subjective judgment and decision making process for patient lifting is discussed [23–25].

One of the most important parameters in the subjective judgment of the healthcare staffs when performing a manual patient handling task is the body mass of prospective patient. Recent studies have shown that as the body mass of the patient increases, the risk of musculoskeletal disorders enhances in the carrier [18, 26, 27]. Having information about the reliability and validity of subjective tools on the topic of patient handling in different countries and cultures can be a great help in assessing nurses’ work situations [20]. These tools are more valuable when they include information such as patient body mass in data collection [19, 28].

The validity and reliability of the two tools (one is especially for super heavy obese patients) for assessing nurses’ perception regards to manual patient handling were addressed and second aim of the current study was to test the following hypothesis:

- H_0_: Nurses’ perception of carrying super heavy and obese patients differs from their perception of carrying normal patients.

## Methods

### Study design, setting, and participants

This cross-sectional study was done between August and December 2019 in the in the educational hospitals of two cities in Iran. In coordination with the nursing team, participants were enrolled from the nurses and other patient handlers. The current study assessed a sample size of 350 participants who performed patient handling in the hospitals. A sample of 350 patient handlers was participated from these hospitals. Patient handlers whom they didn’t accept defined procedures were excluded from the study. The inclusion criterion included all patient handlers who have done patient handling for minimum 1 year. From all, 249 participants were remaining to the end of the research.

### The original instruments

#### Nurses’ attitudes regarding the safe handling of patients who are morbidly obese

This questionnaire includes 26 questions with 5-point Likert (strongly agree to strongly disagree). In addition, a set of demographic questions were asked. At the beginning of the questionnaire, the definitions of super heavy and obese patients were explained and six of 26 items, were asked about obesity (e.g., I believe obesity is due to lack of self-control). The content validity index and test-retest reliability scores were reported as satisfactory by authors. All of 26 items were classified into nine subscales including: Nurses’ perception of stress/ demands of patients’ handling who are morbidly obese, Nurses’ perception of controllable factors of obesity, Nurses’ motivation to use safe handling equipment with patients who are morbidly obese, Nurses’ perception of time/ workload involved in SPH of patients who are morbidly obese, Nurses’ perception of nursing peers’ responses to patients who are morbidly obese, Nurses’ perceived confidence in assessing safe handling needs of patients who are morbidly obese, Nurses’ perception of safety as a priority, Nurses’ perception of uncontrollable factors of obesity, Nurse’s own response to patients who are morbidly obese [19].

#### Safe patient handling perception scale

This tool was introduced by some researchers affiliated to U.S.A. The aim of this 17-item questionnaire was to assess perceptual risk of musculoskeletal disorders in the healthcare context. Seventeen items were grouped into 3 themes according to factor structure analysis: knowledge (11 item), practice (3 item), and resource accessibility (3 item). Alpha score was reported for each subscale which was 0.886, 0.901 and 0.855 for knowledge, practice and accessibility subscales, respectively. The authors stated that this measure can be used to assess employee perceptions of safe patient handling policies and practices. The 5-point Likert scoring was used to gather the data [20].

### Translation procedure

As recommended by literature, linguistic validation technique was used. Translation of the questionnaire from English to Persian was done by two bilingual qualified translators. They had a long experience in occupational health and Ergonomics. Main researcher of the current study prepared the final Persian version of two mentioned translated questionnaires due to the agreement between their similarities. Afterwards, a great specialist of occupational health and Ergonomics, who was blinded about the original version of the questionnaire, translated the Persian version back into English. Finally, this English version was sent to the corresponding author of the main paper that allowed us to utilize their questionnaire and she confirmed the translation [19, 20]. The aim of this step was to ensure that the content is identical to the original one. The original and back-translated versions were checked by the research team of current study completely, and final version of the questionnaire in Persian was prepared.

### Reliability assessment

Reliability of the translated version was assessed by Cronbach’s alpha coefficient which estimates the internal consistency of tools [29]. This procedure was calculated based on item-total correlation and estimate of alpha on which an item was removed from the scale. If 70% of the variance of the observed score was systematic, and the left 30% was due to random errors, the alpha was reported as 70% and considered acceptable.

### Validity assessment

Validity testing for the two scales was based on content validity index/ratio analysis. An expert panel consisting of nurses and other caregivers that doing patient handling tasks [5] and Occupational Ergonomists [5] were participated in this stage. The Lawshe’s method for analysis of Content Validity Ratio (CVR) was used in the present study [30]. Responses of experts for each item were divided into three categories, including: "necessary", "useful, but unnecessary" and "Unnecessary". The CVR calculated according to completed questionnaires as depicted below:

$$\mathrm{CVR}=\frac{ne-{\displaystyle\frac N2}}{\displaystyle\frac N2}$$

ne: number of persons responding to requested questions

N: total number of experts.

Simplicity, relevancy and clarity were considered by experts separately to be scored in evaluating the CVI in a Likert scale Simplicity, relevancy and clarity were considered by experts separately to be scored in evaluating the CVI in a Likert scale.

### Data analysis

Descriptive analyses were carried out to describe the patient handlers’ characteristics. Kolmogorov–Smirnov was used to assess normal distribution of data. After checking the CVR and CVI, for testing the validity of instruments, an Exploratory Factor Analysis was run to extract latent factors [31, 32]. The standard Eigenvalue greater than one and scree plot was used to specify the number of extracted factors. For testing reliability of instruments, Cronbach’s alpha, a measure of internal consistency, was calculated for each sub-component. Kruskal-Wallis Test or Mann–Whitney U was used. Analyses were conducted by SPSS. A significance level of 0.05 was utilized for testing the hypothesis.

### Ethical approval

This study was approved by the ethical committee of Hamadan University of Medical Sciences (Reference: 980210777). The nursing management of each ward was also approved the procedure. Written consent was obtained from all of 249 patient handlers.

## Results

Totally 247 nurses and other patient handlers were participated. Each nurse answered two mentioned questionnaires which one of them was designed for handling of super heavy and obese patients. In this section, descriptive characteristics of participants and the reliability and validity of two questionnaires have presented respectively.

### Description of participants

One hundred seventeen participants were male. Only 36% of participants were nurses and most of them had bachelor’s degree in nursing field. More than 40% of them declared that they had previous musculoskeletal pain. As results have shown in Table 1, more than 85% of them reported their health status as good or moderate and 63% of them had heard about Ergonomics or previously passed a related course. Other descriptive results present in Table 2.

**Table 1:** Description of qualitative parameters of patient handlers

**Variable**	**N**	**Percentage**
**Gender**		
Woman	130	**52.6**
Man	117	**47.4**
**Position**		
Nurse	86	**36.3**
First Aider	41	**17.3**
Assistance	77	**32.5**
Service Personnel	33	**13.9**
**Education**		
Diploma and lesser	47	**20.3**
Upper diploma	70	**30.2**
Bachelor	108	**46.6**
Upper bachelor	7	**3**
**Marital Status**		
Single	97	**41.5**
Married	131	**56**
Divorced	6	**2.6**
**Chronic OR acute MSDs**		
Yes	102	**41.1**
No	146	**58.9**
**Course Ergonomics**		
Yes	159	**64.4**
No	88	**35.6**
**Regular Exercise**		
Yes	127	**51**
No	122	**49**
**Physical Health Status**		
Good	79	**31.7**
Mean	137	**55**
Bad	33	**13.3**

**Table 2:** Description of quantitative demographic parameters of participants

**Variables**	**N**	**Min**	**Max**	**Mean**	**Std. Dev**
**Age**	235	17	72	35.38	11.858
**Experience**	192	1	22	7.02	5.257
**Stature**	166	147	192	163.64	22.454
**Body Mass**	170	61	115	67.85	13.376

### Reliability and validity of two questionnaires

#### Nurses’ attitudes regarding the safe handling of patients who are morbidly obese

**Fig. 1:**
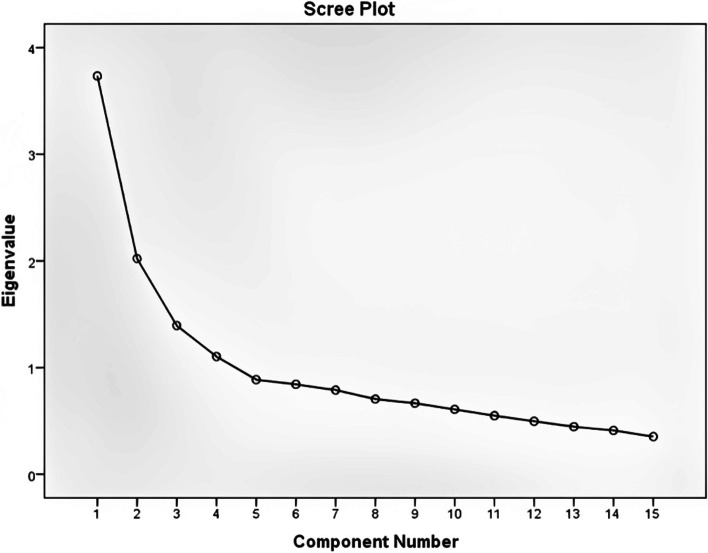
Score plot of remained item in questionnaire

This questionnaire assess was about super heavy and obese patients’ handling. The initial version of this instrument had 26 questions in nine subscales. Calculating content validity index and content validity ratio based on opinion of 4 lay and 5 academic experts, caused 11 items’ deletion (CVR < 0.78). Analyzing factor structure revealed new structure with 4 new components and 15 questions. Due to the acceptable level of Kaiser‐Meyer‐Olkin Measure of Sampling Adequacy (0.777) and Bartlett’s Test of Sphericity (*p*-value < 0.001) 15 remained questions were categorized in four subscales. Score plot of these items depicted in Fig. 1.

The questions, their subscale, and reliability score of the questionnaire were presented in Table 3.

**Table 3:** Results of rotated component matrix

**Questions**	**Components**			
	**1: Experience**	**2: Perception**	**3: Attitude**	**4: Judgment**
Q10: It is important to use lifting equipment for moving a patient with morbid obesity in order to protect myself from injury	**0.759**			
Q13: Workers’ injuries while handling patients can be predicted by using lifting equipment	**0.644**			
Q11: I am confident in assessing the level of assistance needed for patients who are morbidly obese	**0.679**			
Q07: It is time consuming to transfer a patient with morbid obesity from bed to chair by using patient-handling equipment	**0.613**			
Q09: It is time consuming to move or position the patients with morbid obesity by using patient relocation device	**0.499**			
Q06: It is important to use lifting equipment to move patients with morbid obesity	**0.695**			
Q04: If I am given the choice, I would prefer not to take care of patients with morbid obesity		**0.768**		
Q02: Taking care of patients who are obese is stressful for me		**0.659**		
Q03: Taking care of patients with morbid obesity is physically high demanding		**0.582**		
Q05: Many nurses I work with have negative reactions toward patients who are morbidly obese		**0.719**		
Q14: It is often so easier to apply manual handling techniques than using lifting equipment in the case of patients with morbid obesity			**0.785**	
Q08: I often use lifting equipment when working with patients who are morbidly obese			**0.571**	
Q16: In general, staff safety is considered as a priority by the nurses and the managers in my work unit			**0.510**	
Q15: In general, patient safety is considered as a priority by the nurses and the managers in my work unit				**0.798**
Q17: My workload interferes with my ability to use lifting equipment in order to handling patients				**0.592**
**Reliability of Subscales based on Cronbach's alpha coefficient**	**0.766**	**0.780**	**0.781**	**0.736**

#### Safe patient handling perception scale

This instrument initially introduced with 17 items which were categorized in three subscales including: knowledge, Practice and Resource accessibility. In current study 3 items (8, 9 and 13) were excluded based on expert panel team’s opinion (CVR < 0.62). Table 3 illustrates the results of sample adequacy and Sphericity based on KMO and Bartlett’s Tests.

Results of total variances explained in Table 4. Based on the results, a new questionnaire with two subscale and 14 items were introduced for further studies. The reliability score of each subscale was presented in Table 5.

**Table 4:** Results of KMO and Bartlett’s test

**Kaiser–Meyer–Olkin measure of sampling adequacy**		**0.830**
**Bartlett’s Test of Sphericity**	Approx. Chi-Square	1131.314
	df	91
	Sig	** < 0.001**

**Table 5:** Total variance explained for new questionnaire

**Component**	Extraction sums of squared loadings			Rotation sums of squared loadings		
	Total	% of Variance	Cumulative %	Total	% of Variance	Cumulative %
**1**	4.792	34.232	34.232	4.088	29.197	**29.197**
**2**	2.133	15.233	49.464	2.837	20.267	**49.464**

Also, Table 6 illustrates the results of principal Component Analysis with Kaiser Normalization Varimax which proposed new subscales and their related items.

**Table 6:** Results of rotated component matrix

**Questions**	**Component**	
	**1: Work Awareness**	**2: Work Culture**
Q2: I am able to identify the high-risk patient-handling tasks prior to moving a patient	**0.774**	
Q3: Safe patient-handling training prepares me to do patient-handling tasks in my workplace	**0.748**	
Q1: Patient assessments including handling tasks, equipment, space, time and safety concerns	**0.714**	
Q4: Compared to the last year, my job has become more demanding in terms of physical tasks this year	**0.684**	
Q8: I feel comfortable asking my colleagues to help me move a patient	**0.659**	
Q9: I report all patient-handling-related injuries to my supervisors when an injury occurs	**0.591**	
Q6: I understood the policy of the safe patient handling in my work setting	**0.588**	
Q5: I can perform safe patient-handling tasks without hurting myself or patients	**0.550**	
Q10: The content of safe patient-handling training is satisfactory	**0.526**	
Q13: The proper patient handling equipment is accessible		**0.803**
Q14: The patient handling equipment is regularly maintained		**0.742**
Q12: The quality of the patient lifting equipment is satisfactory		**0.726**
Q11: The frequency of safe patient-handling training is satisfactory		**0.686**
Q7: The safe patient handling policy in my complex is accessible to me		**0.455**
**Reliability of Subscales based on Cronbach's alpha coefficient**	**0.836**	**0.748**

### Relationship between demographic variables and the questionnaires

Only stature and body mass from quantitative variables and educational level, official position and passed Ergonomics courses from qualitative variables had significantly correlated by mean score of two questionnaires (Table 7). As the results in Table 8, mean score of the questionnaires are increasing with passing Ergonomics courses, reporting good subjective health status and increasing in education level of participants.

**Table 7:** Correlation between quantitative variables and mean score of questionnaires

**Variable**	**Questionnaire 1**		**Questionnaire 2**	
	**Spearman correlation**	***P*** **-value**	**Spearman correlation**	***P*** **-value**
**Age**	-0.252	0.212	-0.216	0.106
**Work Experience**	0.033	0.645	0.124	0.087
**Stature**	0.303	< 0.001	0.234	0.002
**Body Mass**	0.268	< 0.001	0.17	0.027

**Table 8:** Correlation between qualitative variables and mean score of questionnaires

Variable	Questionnaire 1		Questionnaire 2	
	Z –Score or Chi-Square	*P*-value	Z-Score or Chi-Square	*P*-value
Gender	-0.611	0.541	-0.858	0.391
Chronic OR Acute MSDs	-1.629	0.103	-0.165	0.869
Course Ergonomics	2.295	0.022	3.082	0.002
Doing Regular Exercise	-0.926	0.354	-3.942	< 0.001
Official Position	20.875	< 0.001	15.463	0.001
Education level	19.527	< 0.001	11.201	0.011
Marital Status	1.226	0.542	0.799	0.671
Subjective Health status	19.329	< 0.001	4.563	0.102

### Differences between perception of patient handlers in regards to handling of normal and super heavy patients

Because of abnormality of the data, Wilcoxon Signed Ranks Test used to assess the differences of perception. The 2-tailed assumption was used to investigate this difference. Results showed that patient handlers differentiate between various handling situations of normal and obese patients (Table 9).

**Table 9:** Wilcoxon signed ranks test for analysis of differences between perceptions of patient handlers

Ranks				
		**N**	**Mean rank**	**Sum of ranks**
**Ques1‐Ques2**	Negative Ranks	25^a^	41.92	1048.00
	Positive Ranks	221^b^	132.73	29,333.00
	Ties	1^c^		
	Total	247		
**Sig. (2-tailed)**	***P*** ** < 0.001**			

^a^Ques1 < Ques2

^b^Ques1 > Ques2

^c^Ques1 = Ques2

## Discussion

The aim of the present study was to shed light on the applicability of two well-psychometrically instruments for patient handling and surveying differences between patient handlers’ perception of handling normal and obese patients. Also, the results of re-structural analysis and Persian adopted version are included. According to the results, there was a significant difference between the perception of carrying obese and normal body mass patients.

There is a same point in the literature review that occupational complaints increase whenever nurses and nursing assistants involve in handling of overweight or obese patients [27]. This issue has also stated in ‘Best Practices for Safe Handling of the Morbidly Obese Patient’ [33]. Moreover a recent study by Ugras et al. has demonstrated nurses’ reluctance to move obese patients [34]. Patients’ body mass is considered as a crucial parameter in a newly introduced Risk Index for patient handling [9]. In addition the differences between handling of heavy and normal patients has been considered as a hot topic in other emergency workers [18].

Others results showed that the reliability and validity of Persian adopted version of Safe Patient Handling Perception Scale and Nurses’ Attitudes Regarding the Safe Handling of Patients Who Are Morbidly Obese, were satisfactory. In line with this cross-cultural study, subjective judgment of patient handlers in analysis of their work considered in other studies [28, 35, 36], and cross-cultural studies are common in examining the nurses’ work environment [37]. Acceptable level of validity and reliability scores were similar to other studies [38, 39]. It was possible to remove questions due to low reliability in cross cultural assessment of patient handling and patient transfer studies [40, 41].

Alongside all other relevant studies, reliability of Persian version of Safe Patient Handling Perception Scale was remained acceptable such as its original version [20]. To the best of our knowledge, other translated versions of mentioned questionnaire in current study were not introduced yet. Also the Persian version of Nurses’ Attitudes Regarding the Safe Handling of Patients Who Are Morbidly Obese also had a good reliability score like its Original version [19].

The limitation of the current study was the sample size, which may have influenced the power of the study. It’s recommended to increase the acceptability of theses questionnaires in future studies for other cultures.

## Conclusions

These two instruments evaluated for manual handling of normal and obese patients had acceptable validity and reliability among Iranian Nursing. So they can be used in further studies with the same cultures to assess patient handling task from patient handlers’ point of view.

## Data Availability

All data generated or analyzed during this study are included in this published article. Also, more information can be asked on the reasonable request from corresponding author.
